# Dietary Vitamin D Supplementation Improves Hair Follicle Development and Affects Fatty Acid Metabolism in Rex Rabbits

**DOI:** 10.1002/fsn3.70675

**Published:** 2025-07-21

**Authors:** Xiang Li, Xiaojing Wu, Fuchang Li, Lei Liu

**Affiliations:** ^1^ Key Laboratory of Efficient Utilization of Non‐Grain Feed Resources (Co‐Construction by Ministry and Province), Ministry of Agriculture and Rural Affairs, Shandong Provincial Key Laboratory of Animal Nutrition and Efficient Feeding, Department of Animal Science Shandong Agricultural University Taian Shandong China

**Keywords:** fat metabolism, hair follicle development, Rex rabbits, vitamin D

## Abstract

An experiment was conducted to investigate the effect of different levels of vitamin D (VD) supplementation in rabbit diets on hair follicle development and fatty acid metabolism. Two hundred Rex rabbits with similar body weight were divided randomly into five groups (40 replicates per group and 1 rabbit per replicate): control group fed a basic diet; experimental groups fed respectively a basic diet with an addition of 700, 1400, 2100, or 2800 IU/kg VD. Dietary addition of 2800 IU/kg VD significantly increased hair length (*p <* 0.05). Dietary addition of 700–2100 IU/kg VD significantly decreased average hair fineness (*p <* 0.05). Dietary addition of 1400–2800 IU/kg VD significantly increased total hair follicle density and secondary hair follicle density (*p <* 0.05). Dietary addition of VD (2100 IU/kg) significantly increased the mRNA levels of Wnt10b, β‐catenin, insulin‐like growth factor 1 (IGF1), epidermal growth factor (EGF), sonic hedgehog (SHH), and Notch in rabbit skin (*p <* 0.05). Dietary addition of VD (2100 IU/kg) significantly increased the mRNA levels of Carnitine palmitoyl transferase (CPT1) 1 and CPT2 in fat, CPT1, CPT2, and peroxisome proliferator‐activated receptor‐α in liver, and fatty acid‐transport protein and fatty acid‐binding protein in skeletal muscle, but significantly decreased the mRNA level of acetyl coenzyme A carboxylase in fat (*p <* 0.05). Dietary VD addition promotes hair follicle development. Wnt10b/β‐catenin, Notch, SHH, IGF1, and EGF signaling pathways or factors may participate in the regulatory process. Dietary VD addition increases the fatty acid oxidation process in fat tissue and liver, and fatty acid uptake in muscle, and inhibits the fatty acid synthesis process in fat tissue. Dietary addition of 1400–2100 IU/kg VD is a better appropriate dose for hair density for Rex rabbits.

## Graphical abstract

This article discusses the effects of adding different concentrations of vitamin D to the diet on the development of hair follicles in rex rabbits and on lipid metabolism in their muscle, liver, and fat tissues.

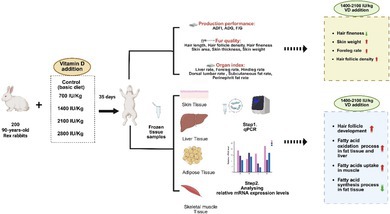

## 1 Introduction

Hair follicles represent intricate dermal appendages characterized by a distinctive physiological architecture and cyclic growth patterns. They undergo a continuous cycle comprising three distinct phases: anagen (growth), catagen (regression), and telogen (rest) (Lin et al. 2022). This repetitive cycle persists throughout the entire lifespan of rabbits. Besides, these processes are regulated by many factors or signaling pathways. SHH, Noggin, EGF, IGF1, and Wnt10b/β‐catenin can improve hair follicle development (Rishikaysh et al. 2014). Bone morphogenetic proteins (BMPs) and transforming growth factor β (TGFβ) can inhibit hair follicle development. MicroRNAs are also an important regulating factor in hair follicle development (Yang et al. 2004). MiR‐205 can promote hair regeneration by improving the mechanical properties of hair follicle stem cells (Wang et al. 2023).

Hair follicle development can be affected by diet nutrient composition and nutrient level (Guo and Katta 2017). Vitamin D (VD) is a steroid hormone produced in epidermal keratinocytes when exposed to UV‐B light (290–315 nm) or obtained through dietary intake and supplements (Nayak et al. 2016). VD is vital for maintaining epidermal homeostasis. The previous study found that VD could maintain the proper function and survival of the keratinocyte stem cell population in the bulge region of hair follicles, which is essential for hair growth and overall skin health (Banihashemi et al. 2016). Absence of the VD receptor (VDR) stops the self‐renewal of the epidermal keratinocyte stem cell population, resulting in the absence of post‐morphogenic hair cycles (Demay 2012). The use of oral VD_3_ significantly improves hair regrowth in patients with telogen effluvium (Sattar et al. 2021). Although there is no direct evidence that excessive VD intake has a negative effect on the skin and hair follicles, excessive VD intake triggers abnormalities in calcium metabolism, leading to hypercalcemia and hypercalciuria, which is not good for skin health and hair follicle development (Frieder et al. 2018). The molecular partners or downstream target genes of VD in regulating hair growth have not yet been identified.

Fatty acids maintain animal health through the multidimensional aspects of energy supply, structural function, metabolic regulation, and immunomodulation, and their types and sources have significant differences in physiological effects. Long‐chain fatty acids serve as high‐density energy substances that provide sustained energy to animals. It has been found that the addition of fats and oils to rations significantly increases energy density and supports the realization of genetic potential in high‐performance livestock (Yin and Lai 2022). In addition to directly supplying fatty acids, they could regulate immune cell activity and inflammatory factor secretion, which affected the skin barrier function and hair follicle development (Cheng and Kong 2021). Dietary VD deficiency influenced fatty acid desaturase activity and expression, and therefore altered fatty acid metabolism (Nandi et al. 2019).

VD can also regulate the process of lipid metabolism (Ding et al. 2012). Adipose tissue is the main storage site of VD; the different forms of VD affect the stages of adipogenesis, adipocyte differentiation, and apoptosis. The expression of VDR was observed during the initial stages of adipocyte differentiation (Fu et al. 2005). VDR could inhibit adipogenesis (Kong and Li 2006). VD addition could activate VDR and promote fatty acid β‐oxidation to supply energy. 1α‐25(OH)_2_‐D_3_ induces the activation of VDR, which subsequently forms a heterodimeric complex with retinoid X receptor (RXR). This transcriptional complex binds to VD response elements (VDREs) located in target gene promoter regions, mediating ligand‐dependent transcriptional regulation. The activated signaling cascade promotes the upregulation of fatty acid β‐oxidation while suppressing lipogenic pathways (Sabir et al. 2017). 1α‐25(OH)_2_‐D_3_ exerted an anti‐adaptogenic effect via the Wnt/β‐catenin pathway (Lee et al. 2012) and inhibited adipocyte differentiation via the extracellular regulated kinase pathway (ERK) (Sakuma et al. 2012). It has been demonstrated that low doses of 1α‐25(OH)_2_‐D_3_ inhibit apoptosis in differentiated 3T3‐L1 cells, whereas high doses stimulate apoptosis (Sun and Zemel 2004). VD has an inhibitory effect on body fat mass via inhibiting the preadipogenic differentiation process (Sharma et al. 2014). VD_3_ supplementation led to body fat mass reduction in healthy overweight and obese women (Salehpour et al. 2012). The preceding study demonstrated that VD_3_ supplementation (100 nm for 5 days) inhibited foam cell formation by reducing cholesterol deposition and enhancing cholesterol efflux in human monocyte‐derived macrophages from adults with type 2 diabetes mellitus (T2DM) (Marino et al. 2022). Chang and Kim (2016) documented that VD_3_ treatment (100 nm for 24 h) decreased lipid accumulation and increased lipolysis in 3T3‐L1 cells. Marino et al. (2022) demonstrated that VD_3_ treatment could counteract lipid storage by 79% compared to the control in 3T3‐L1 cells.

In the present study, we investigate the effect of the addition of VD in rabbit diet on hair follicle development, lipid metabolism, and related factors or signaling pathways, and determine the possible mechanism of VD regulating hair follicle development and lipid metabolism. Our results clarified the regulatory effect and the mechanism of VD in hair follicle development and lipid metabolism, and also revealed the appropriate addition level of VD in the diet of growing Rex rabbits.

## 2 Materials and Methods

### 2.1 Animal Care

All study procedures were approved by the Shandong Agricultural University Animal Care and Use Committee (SDAUA‐2021‐105) and were in accordance with the Guidelines for Experimental Animals established by the Ministry of Science and Technology (Beijing, China).

### 2.2 Animals, Diets, and Experimental Design

A total of 200 mixed‐sex Rex rabbits were individually housed in custom‐designed cages measuring 60 × 40 × 40 cm. Temperature and lighting conditions were maintained in accordance with commercial standards. The diets in our study were pressure‐pelleted, with a pellet diameter of 4 mm. The ingredients and nutrient composition of the basal diet are presented in Table 1. All rabbits had free access to feed and water during the rearing period. Two hundred Rex rabbits (90‐day‐old) with similar body weight (1720 ± 12 g) were divided into 5 groups (40 replicates per group and 1 rabbit per replicate): control group fed basic diet; experimental groups fed a basic diet with an addition of 700, 1400, 2100, or 2800 IU/kg VD. VD level was determined according to the rabbit's requirement and the previous study (Lani et al. 2014). The pre‐experiment lasted 3 days, and the trial period was 35 days.

**TABLE 1:** Composition and nutrient levels of basal diets (air dry basis) %.

Ingredients	Content	Nutrient levels^a^	Content
Corn	8.2	Digestible energy/(MJ/kg)	10.00
Wheat shorts	5	Dry matte	88.77
Wheat bran	18	Crude protein	14.52
Germ	12	Ash	7.02
CP14% Alfalfa meal	15	Ether extract	3.51
Sunflower meal	11.5	Crude protein	20.26
Soybean meal	6	Calcium	0.75
Peanut shell	13	Phosphorus	0.43
Husk powder	6		
Bean oil	1.3		
Limestone	1.1		
70% lysine	0.5		
Premix^b^	2.4		
Total	100		

*Note:* Ingredients, proximate composition were measured and analyzed based on triplicate determination.

^a^ Nutrient levels were calculated value.

^b^ Premix/kg: Vitamin A, 10,000 IU; Vitamin E, 60 mg; Vitamin K, 32 mg; Vitamin B_1_, 5 mg; Vitamin B_2_, 10 mg; Vitamin B_11_, 2.5 mg; Vitamin B_12_, 0.01 mg; Choline chloride, 120 mg; Fe (ferrous sulfate), 50 mg; Zn (zinc), 50 mg; Mn (manganese sulfate), 3 mg; Se (selenium), 0.4 mg; I (as iodine), 0.6 mg; Cu (copper sulfate), 30 mg; CaHPO_4_, 6000 mg; NaCl, 5000 mg; Met 99%, 1500 mg.

### 2.3 Data and Sample Collection

Feed consumption and body mass were measured once a week. At the end of the trial, eight rabbits from each group were selected to collect samples. Rabbit hairs were gathered from the abdomen, hip, shoulder, and back. Blood samples were collected directly from the heart of each rabbit using a sterile syringe under appropriate anesthesia. Plasma was then obtained following centrifugation at 400 *g* for 10 min at 4°C and stored at −20°C for subsequent analysis. Cervical dislocation was used to sacrifice the rabbits, and the liver, dorsal lumbar muscle, foreleg and hindleg muscles, subcutaneous fat, and perinephric fat were gathered and weighed. Following snap‐freezing in liquid nitrogen, the tissue samples were stored at −80°C for subsequent RNA analyses. The samples of mediodorsal skin were collected for Hematoxylin–Eosin (HE) staining. Additionally, hair follicle cells were isolated from another mediodorsal skin sample using a previously published method (Weinberg et al. 1993), then frozen in liquid nitrogen and stored at −80°C for further RNA analyses.

### 2.4 Measurements

The length of rabbit hairs from the shoulder, back, hip, and abdomen was measured using a ruler. Wool fiber diameter was measured using an electronic fiber fineness detector (model PS‐W‐4, Shanghai Juhong Instrument Equipment Co. Ltd. Shanghai, China). A soft ruler was used to measure the width and length of rabbit skin at the narrowest point. Then, the area of rabbit skin was calculated. A vernier caliper was used to measure skin thickness.

The skin samples were processed, embedded, and stained according to the procedure outlined by Yue et al. (2022). Briefly, the skin samples were fixed in 4% paraformaldehyde, dehydrated, and embedded in paraffin. The sections were stained with HE, then analyzed under a microscope to calculate hair follicle density.

RNA extraction and determination of gene expression were performed using the method of Liu et al. (2017). The total RNA was extracted from the appropriate skin, fat, skeletal muscle, and liver tissue using the Trizol RNA extraction, and then transcribed into cDNA using the reverse transcription kit. The cDNA was subjected to fluorescent quantitative PCR using SYBR Green dye to determine the mRNA expression level of each gene. The sequences of primers are shown in Table 2. The following steps were used in the qPCR reaction: 95°C for 30 s, 1 cycle, 95°C for 5 s, 60°C for 30 s, 40 cycles. The relative expression levels of target genes were calculated by the $2^{-∆∆C_{t}}$ method. Target gene mRNA levels were standardized against those of glyceraldehyde 3‐phosphate dehydrogenase (GAPDH). Analysis based on *C*_t_ values indicated that GAPDH mRNA levels remained consistent across all treatments in this study (*p >* 0.1).

**TABLE 2:** Gene‐specific primers of related genes.

Gene	Genebank accession number	Primers sequences(5′→3′)	Product size (bp)
*GAPDH*	NM_001082253	F: TGCCACCCACTCCTCTACCTTCG	118
		R: CGAAGGTAGGGATGGGTGGCA	
*PPARα*	XM_002723354	F: AGGCCCTCTTCAGAACCTGT	122
		R: GTGGCTTTCTGTTCCCAGAG	
*PPARγ*	NM_001082148.1	F: GGAGCAGAGCAAAGAAGTCG	111
		R: CTCACAAAGCCAGGGATGTT	
*FATP*	XM_002722970	F: GGCCTACCTCTCTGGTGATG	111
		R: TCAGTGGTGGACACGTTCTC	
*FABP*	XM_002716060	F: AGCTGGTGGACAGCAAGAAT	129
		R: TCAGGGTGATGATGTCTCCA	
*CPT1*	XM_002724092.2	F: ATTCTCACCGCTTTGGGAGG	196
		R: ACGGGGTTTTCTAGGAGCAC	
*CPT2*	XM_008265231.1	F: ATGACCGTTTCTGCCATCC	101
		R: AAGGTGTTGGTGTCGCTTCT	
*FAS*	KF201292.1	F: ACCACGTCCAAGGAGAGCA	112
		R: AGTTCTGCACCGAGTTGAGC	
*ACC*	XM_002719077.2	F: GTGGTCTTCGTGTGAACTGG	122
		R: TTCTTCTGCTGCCTTTAGCC	
*HSL*	XM_008249691.2	F: CCAGGCTAAACTCGCATCCA	119
		R: ATTTGGCTCTCTGGACTGGC	
*LPL*	NM_001177330.1	F: TTCAACCACAGCAGCAAGAC	141
		R: TAACAGCCAGTCCACCACAA	
*β‐catenin*	DQ786777.1	F: TTCTTGGGACTCTTGTTCAGC	122
		R: CACTTGGCACACCATCATCT	
*Wnt10b*	NM_002711076	F: TGTGCCATCCCTCTTCCTTA	150
		R: GGCTCCACCTCTAACTTCTGC	
*Notch1*	XM_011518717	F: TGCGAGACCAACATCAACGAGTG	94
		R: TCAGGCAGAAGCAGAGGTAGGC	
*BMP2*	XM_001082650	F: GACATCCTGAGCGAGTTCGAGTTG	113
		R: CGGCGGTACAAGTCCAGCATG	
*BMP4*	NM_001195723	F: CTAAGCATCACCCACAGCGG	163
		R: AGTCATTCCAGCCCACGTC	
*TGFβ‐2*	NM_008249704	F: CCGTTTCTTTCGTGGGATAC	108
		R: GGTAAGGGAGGAGGGTCTCA	
*IGF‐1*	NM_001082026	F: TCTGAGGAGGCTGGAGATGT	122
		R: TGTTGGTAGATGGAGGCTGA	
*EGF*	XM_017347349	F: CCTTCACAGAGCCGATCTCAATGG	153
		R: TCACAAGAGCACATACGAGCACTG	
*Noggin*	XM_002719279	F: CCAGCACTACCTCCACATCC	123
		R: GCGTCTCGTTCAGATCCTTC	
*SHH*	XM_002715022	F: ACGGCCACCATTCGGAGGAG	83
		R: GTACTTGCTACGGTCACGGTCAG	

Abbreviations: *ACC*, acetyl coenzyme A carboxylase; *BMP2*, bone morphogenetic protein 2; *BMP4*, bone morphogenetic protein 4; *CPT1*, Carnitine Palmitoyltransferase 1; *CPT2*, Carnitine Palmitoyltransferase 2; *EGF*, epidermal growth factor; *FABP*, fatty acid binding protein; *FAS*, fatty acid synthase; *FATP*, fatty acid transport protein; *GAPDH*, glyceraldehyde 3‐phosphate dehydrogenase; *HSL*, hormone‐sensitive lipase; *IGF‐1*, insulin‐like growth factor‐1; *LPL*, lipoprotein lipase; *Notch1*, notch homolog 1; *PPARα*, peroxisome proliferator‐activated receptor‐α; *PPARγ*, peroxisome proliferator‐activated receptor‐γ; *SHH*, sonic hedgehog; *TGFβ‐2*, transforming growth factor beta; *Wnt10b*, wnt family member 10b.

### 2.5 Statistical Analysis

All data were subjected to analysis using the general linear model (GLM) procedure of the statistical software SAS. The significance of the differences among the treatments was evaluated using Duncan's multiple range test, and the results were considered to be statistically significant at *p* < 0.05.

## 3 Results

The effect of VD on the production performance of Rex rabbits was shown in Table 3. Dietary addition of 2100 IU/kg VD significantly decreased average daily feed intake (*p <* 0.05). Dietary addition of VD had no significant effect on average daily body weight gain, feed conversion efficiency, skin area, and skin thickness of rabbits (*p >* 0.05), but significantly increased skin weight (*p <* 0.05). Dietary addition of 2800 IU/kg VD significantly increased hair length (*p <* 0.05). Dietary addition of 700–2100 IU/kg VD significantly decreased average hair fineness (*p <* 0.05).

**TABLE 3:** Effects of dietary VD levels on production performance of Rex rabbits.

Items	Dietary vitamin D addition level (IU/kg)				
	0	700	1400	2100	2800
Average daily feed intake (g)	180.2 ± 12.7^a^	180.3 ± 10.3^a^	174.4 ± 20.6^ab^	168.9 ± 14.6^b^	177.2 ± 12.4^a^
Average daily gain (g)	27.06 ± 4.69	27.80 ± 4.97	26.71 ± 5.47	26.41 ± 5.32	27.30 ± 5.82
Feed/gain	6.82 ± 1.04	6.66 ± 1.07	6.68 ± 1.00	6.59 ± 1.12	6.73 ± 1.24
Hair length (cm)	2.20 ± 0.089^b^	2.2 ± 0.192^b^	2.18 ± 0.088^b^	2.20 ± 0.113^b^	2.38 ± 0.148^a^
Hair fineness (μm)	17.30 ± 0.63^a^	16.48 ± 1.05^bc^	16.08 ± 0.58^cd^	15.46 ± 0.77^d^	17.19 ± 0.63^ab^
Skin area (cm^2^)	1422.06 ± 79.73	1463.67 ± 50.75	1467.45 ± 21.07	1460.35 ± 70.18	1481.66 ± 39.12
Skin thickness (mm)	1.17 ± 0.16	1.16 ± 0.24	0.96 ± 0.32	0.91 ± 0.26	1.00 ± 0.15
Skin weight (g)	329.50 ± 99.73^b^	408.8 ± 29.45^a^	402.75 ± 26.56^a^	392.00 ± 53.42^a^	399.62 ± 29.68^a^

*Note:* In the same row, values with no letter or the same letter superscripts mean no significant difference (*p* > 0.05), while those with different small letter superscripts mean a significant difference (*p* < 0.05), (*n* = 8).

The effects of dietary VD addition on indices of tissue and organ of Rex rabbits were shown in Table 4. Dietary addition of 1400–2100 IU/kg VD significantly increased foreleg rate (*p <* 0.05). Dietary addition of 1400–2800 IU/kg VD significantly increased hindleg rate (*p <* 0.05). Dietary addition of different levels of VD did not significantly affect liver rate, dorsal lumbar rate, subcutaneous fat rate, and perinephric fat rate (*p >* 0.05).

**TABLE 4:** Effects of dietary VD on indices of tissue and organ of Rex rabbits.

Items (g/kg)	Dietary vitamin D addition level (IU/kg)				
	0	700	1400	2100	2800
Liver rate	2.477 ± 0.29	2.425 ± 0.26	2.42 ± 0.37	2.699 ± 0.31	2.439 ± 0.25
Foreleg rate	6.06 ± 0.58^c^	6.41 ± 0.478^bc^	7.63 ± 0.53^a^	6.798 ± 0.45^b^	6.49 ± 0.41^bc^
Hindleg rate	15.32 ± 0.85^b^	16.19 ± 0.95^ab^	16.40 ± 1.18^a^	16.39 ± 0.62^a^	16.38 ± 0.57^a^
Dorsal lumbar rate	4.679 ± 0.44	4.84 ± 0.32	4.86 ± 0.44	4.44 ± 0.29	4.75 ± 0.25
Subcutaneous fat rate	0.51 ± 0.11	0.45 ± 0.17	0.31 ± 0.10	0.42 ± 0.15	0.45 ± 0.11
Perinephrit fat rate	1.431 ± 0.435	2.161 ± 0.480	1.81 ± 0.561	1.870 ± 0.553	1.62 ± 0.75

*Note:* In the same row, values with no letter or the same letter superscripts mean no significant difference (*p* > 0.05), while those with different small letter superscripts mean significant difference (*p* < 0.05), (*n* = 8).

**FIGURE 1:**
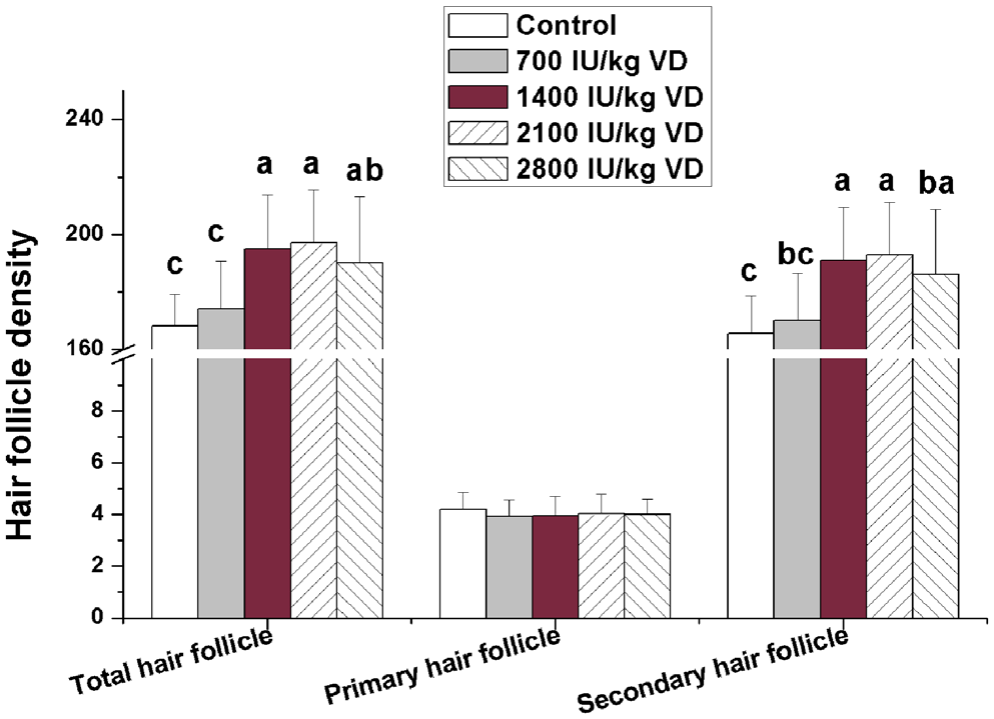
Effects of dietary VD addition on hair follicle density in rabbit skin (*n* = 8). Comparison of total hair follicle density, primary hair follicle density, and secondary hair follicle density in otter rabbits with different VD additions, where different letters indicate significant differences (*p* < 0.05).

**FIGURE 2:**
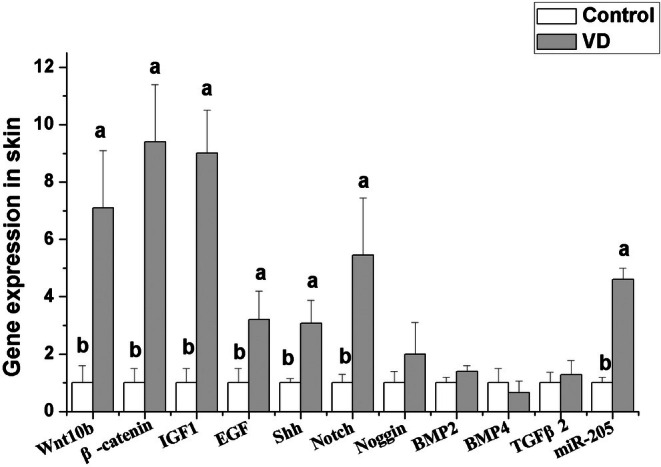
Effects of dietary VD addition (2100 IU/kg) on gene expression related to hair follicle development in rabbits (*n* = 8).

Number of hair follicles was counted after HE staining of skin tissue. The results were shown in Figure 1. Dietary addition of 1400–2800 IU/kg VD significantly increased the total hair follicle density and secondary hair follicle density (*p <* 0.05). Dietary addition of different levels of VD did not significantly affect the primary hair follicle density (*p >* 0.05). According to the results of production performance and hair follicle density, rabbits fed 2100 IU/kg VD treated group showed the best performance. So, we selected the control group and 2100 IU/kg VD‐treated group to detect the gene expression related to hair follicle development in skin and fat metabolism in liver, fat, and skeletal muscle. The effects of dietary addition of 2100 IU/kg VD on gene expression related to hair follicle development in skin are shown in Figure 2. The results show that dietary addition of 2100 IU/kg VD significantly increased the mRNA levels of Wnt10b, β‐catenin, insulin‐like growth factor 1 (IGF1), epidermal growth factor (EGF), Sonic hedgehog (SHH) and Notch in rabbit skin (*p* < 0.05), but did not significantly affect the mRNA levels of noggin, bone morphogenetic protein (BMP) 2, BMP4, and transforming growth factor β2 (TGFβ2) (*p >* 0.05).

**FIGURE 3:**
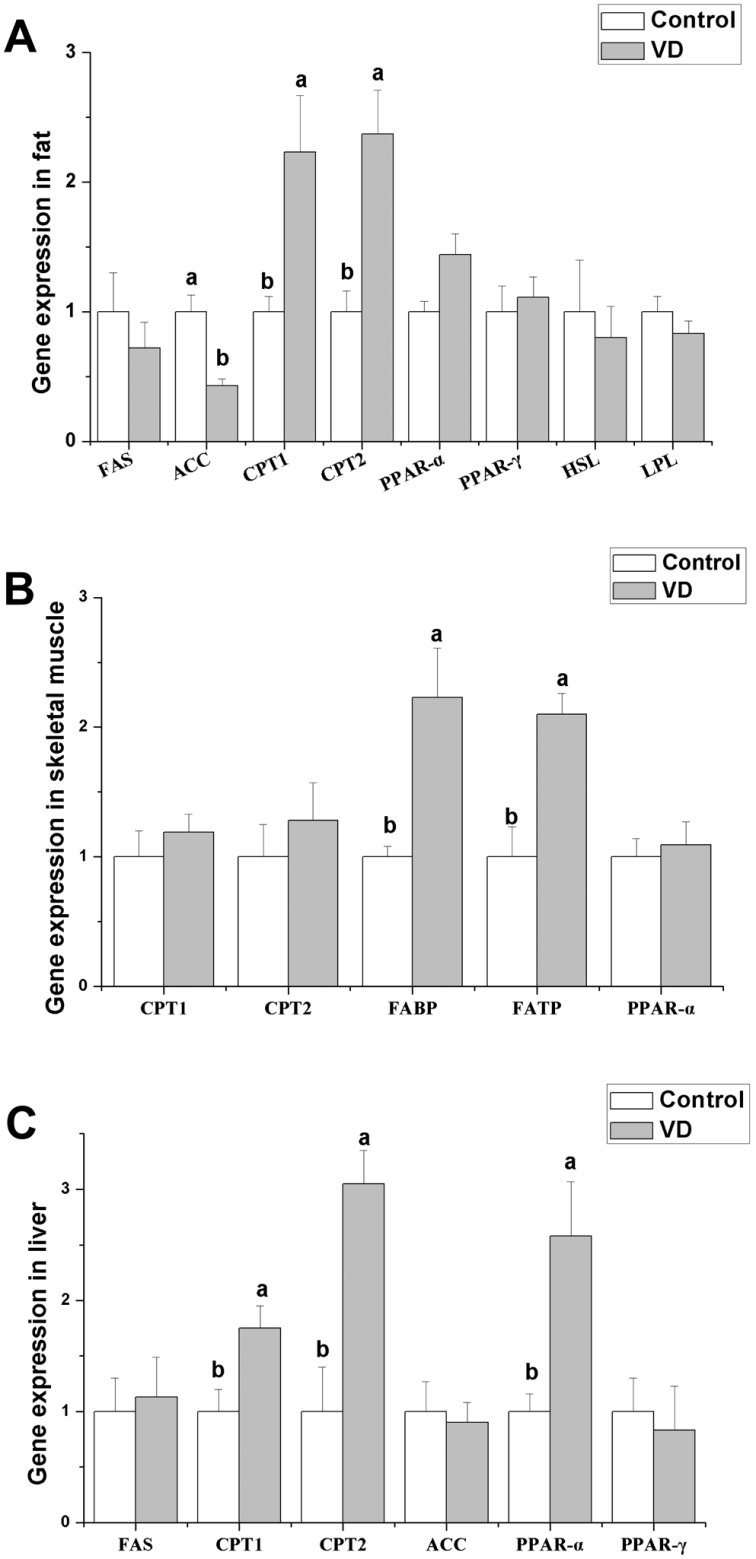
Effects of dietary VD addition (2100 IU/kg) on gene expression related to fat metabolism in rabbits (*n* = 8).

The effects of dietary addition VD (2100 IU/kg) on gene expression related to fat metabolism is shown in Figure 3. Dietary addition of VD increased significantly the mRNA levels of carnitine palmitoyl transferase (CPT1) 1 and CPT2 in fat (*p <* 0.05), but decreased significantly the mRNA level of acetyl coenzyme A carboxylase (ACC) (*p <* 0.05). Dietary addition of VD did not significantly affect mRNA levels of fatty acid synthase (FAS), peroxisome proliferator‐activated receptor (PPAR) α, PPAR‐γ, hormone‐sensitive lipase (HSL) and lipoprotein lipase (LPL) in fat (*p >* 0.05). Dietary VD addition significantly increased the mRNA levels of fatty acid‐transport protein (FATP) and fatty acid‐binding protein (FABP) in skeletal muscle (Figure 3B, *p <* 0.05), but did not significantly affect the mRNA levels of CPT1, CPT2, and PPAR‐α (*p >* 0.05). In liver, dietary addition of VD significantly increased the mRNA levels of CPT1, CPT2, and PPAR‐α (Figure 3C, *p <* 0.05), but did not significantly affect the mRNA levels of FAS, ACC, and PPAR‐α (*p >* 0.05).

## 4 Discussion

### 4.1 Dietary VD Addition Improves Hair Follicle Development

Hair density is a key indicator for judging the quality and economic value of Rex rabbit skin. VD_3_ plays a protective role in radiation‐induced alopecia (Baltalarlı et al. 2006). The previous studies have shown that topical calcitriol (the active form of VD) promotes the regeneration of hair follicle stem cells in mice after chemotherapy‐induced alopecia accelerates (Chen et al. 1998). In the present study, dietary VD addition (1400–2800 IU/kg) increased the total follicle density and secondary follicle density, indicating that VD could promote hair follicle development in consistency with the previous studies (Liu et al. 2021), and the effective dose range is relatively large. 2800 IU/kg VD addition still has not shown a significant negative effect on hair follicle development. But 700 IU/kg VD addition did not have significant effect on follicle density, indicating that the addition dosage of 700 IU/kg VD is insufficient for hair follicle development in Rex rabbits. The previous study in chicks showed that dietary 500 IU/kg VD addition could meet the production demands (Wei et al. 2024). These results suggest that the required dose of VD varied among different species.

Wnt/β‐catenin signaling pathway, as an essential trigger for the initiation of hair follicle matrix formation, can facilitate the transition of hair follicle stem cells from telogen phase to anagen phase (Yue et al. 2022). After binding to VDR, VD can regulate the expression of upstream factors of Wnt (e.g., dikkopf1 and secreted frizzled related protein) and directly affect the Wnt signaling (Pendas‐Franco et al. 2008). Besides, VDR can also interact with β‐catenin and affect the process of β‐catenin entering the nucleus (Hu et al. 2014). VD can regulate many biological processes via affecting Wnt/β‐catenin signaling pathway. Administration of VD could attenuate osteoclastic activity via Wnt/β‐catenin signaling pathway (Xu et al. 2022). VD has been extensively employed in the therapeutic management of vitiligo. In this context, VD could alleviate oxidative stress to protect human melanocytes by activating the Wnt/β‐catenin signaling pathway (Tang et al. 2018). Dietary VD addition also activates the Wnt/β‐catenin signaling pathway by increasing the gene expression of Wnt and β‐catenin, suggesting that Wnt/β‐catenin signaling pathway is a key target of VD promoting hair follicle development. But these results are inconsistent with the study in melanoma of mice, indicating that elevated VD‐VDR signaling inhibited Wnt/β‐catenin signaling gene expression (Muralidhar et al. 2019). The inconsistent results indicate that the differences in VD physiological characteristics and metabolic pathways between species or tissues.

Previous research found that epithelial activation of SHH signaling facilitates hair regeneration (Lim et al. 2018). Dermal SHH signaling regulates specific dermal papillae (DP) signatures to maintain DP maturation and drive hair follicle morphogenesis (Woo 2012). In our study, dietary VD addition significantly increased the gene expression of SHH, indicating that VD can activate the SHH signaling pathway, which is also associated with hair follicle development. Our result is consistent with the previous study indicating that VD could improve cerebral perfusion and reduce neurological impairment in rats via activating the SHH signaling pathway (Bao and Yu 2018). In addition, extensive research has demonstrated that SHH expression is modulated by Wnt signaling pathways. This regulatory mechanism involves the downregulation of E‐cadherin through the action of Lef1, ultimately leading to an uptick in SHH expression levels (St‐Jacques et al. 1998). In this study, the simultaneous increase of Wnt10b and SHH gene expression after VD treatment indicates that the regulation of VD in SHH signaling may be through Wnt10b signaling.

IGF1 has an anti‐apoptotic function, which is critical for hair follicle development. Studies in mice have shown that overexpression of IGF1 in skin could promote hair follicle development (Bol et al. 1997). There was a correlation between VD and IGF1 levels. VD dosages of < 1000 IU/day significantly raised serum IGF1 levels (Kord‐Varkaneh et al. 2020). In this study, dietary VD addition significantly increased IGF1 gene expression in skin, which is similar to VA function in that the supplementation of VA to the diet notably mitigated the suppressive impact of heat stress on IGF1 and IGF1R. These findings suggest that VD may facilitate hair follicle development involving IGF1 signaling.

Evidence has demonstrated that microRNAs play a regulatory role in the development of hair follicles. Specifically, miR‐205 exhibits elevated expression levels in epithelial progenitor cells and stem cells during the developmental stages of mammalian skin (Yi et al. 2008). miR‐205 has been demonstrated to influence hair follicle development by stimulating the proliferation of hair follicle stem cells. Conversely, the absence of miR‐205 impeded the proliferation of hair follicle stem cells and their progeny (Yu et al. 2008). Numerous studies found that VD intake shows protective benefits by modulating miRNAs expression (St‐Jacques et al. 1998). VD can regulate glycoprotein metabolism in swine lung via altering the expression of miR‐205 (Wierzbicka et al. 2024). In our study, dietary VD addition significantly increased the gene expression of miR‐205 in line with the previous study (Xia et al. 2022). These results demonstrate that miR‐205 plays an important role in VD regulating hair follicle development.

Notch signaling pathway is involved in epidermal appendage formation. Notch receptors and their ligands appear in epidermal appendages (Powell et al. 1998). Studies in old Wistar rats show that Notch activity is closely related to muscle regeneration. VD deficiency may exacerbate the age‐related decline in muscle regeneration capacity (Domingues‐Faria et al. 2014). Currently, there is little information about the regulatory effect of VD on Notch signaling pathway in skin or hair follicle. VD treatment significantly protected against dextran sodium sulfate‐induced ulcerative colitis via improving the protein expression of Notch in the guinea pig intestinal mucosa (Qiu et al. 2021). In the present study, dietary VD addition significantly increased the gene expression of Notch in rabbit hair follicle, indicating that Notch signaling pathway is involved in the regulation of VD in hair follicle development.

Epidermal growth factor (EGF) also plays a critical role in the regulation of hair follicle growth and development. Knockdown of the EGF receptor gene resulted in a reduction in hair follicle formation and the promotion of the transition from the anagen phase to the regression phase (Richardson et al. 2009). The previous study showed EGF receptor‐VDR cross‐talk in colitis‐associated colon cancer (Dougherty et al. 2014). In this study, dietary VD addition upregulated EGF gene expression significantly, indicating that dietary VD can activate EGF signaling to regulate hair follicle development in Rex rabbits.

The growth cycle of hair follicles is modulated by various signaling pathways and factors. Bone morphogenetic proteins (BMPs), produced by dermal papillary fibroblasts throughout the telogen phase, are vital in modulating the development, cellular proliferation, differentiation, and apoptosis of both skin and hair follicles (Botchkarev 2003). In contrast, noggin is a high‐efficiency inhibitor of BMP members, binding to BMP4 to directly antagonize and abolish BMP4 activity (Groppe et al. 2002). The upregulation of the noggin gene prolonged the growth phase in hair follicle growth cycles (Botchkarev 2003). Additionally, TGF‐β is involved in the process of hair follicle development, which can suppress the proliferation of keratinocytes (Groppe et al. 2002). In the present study, dietary addition of VD did not significantly affect the gene expression of BMP2, BMP4, noggin, and TGF‐β2, indicating that BMP, noggin, and TGF‐β2 may not be the major target factors in VD regulating hair follicle development in Rex rabbits.

### 4.2 Dietary VD Addition Affects the Process of Fatty Acid Metabolism in Rabbits

Previous research suggests that VD regulates gene expression involved in cell metabolism. Mice with VDR deficiency exhibit resistance to weight gain induced by a high‐fat diet, indicating that VD plays a critical role in regulating global energy metabolism (Fraser 2015). Dietary VD deficiency influences fatty acid composition (Nandi et al. 2019). In the present study, we detected fatty acid metabolism‐related gene expression in skeletal muscle, adipose tissue, and liver.

It has been suggested that VD could play a role in anti‐obesity. VD supplementation in overweight individuals (human and murine) exerts beneficial influences on their energy balance and metabolic homeostasis (Bouillon et al. 2014). Contrary to prior research, our current investigation reveals that VD supplementation does not affect lipid accumulation and gene expression of key enzymes in fat, e.g., HSL, which is pivotal for the hydrolysis of cholesteryl esters and diacylglycerol within adipose tissue, as well as LPL, a central player in lipid and lipoprotein catabolism. In addition, VD administration had no significant impact on the expression of PPARs, crucial transcription factors governing lipid metabolic pathways. These findings suggest that the regulatory effect of VD on fat metabolism exhibits species‐specific variations. In adipose tissue, the present results showed that the rate‐limiting enzymes of fatty acid oxidation of CPT1 and CPT2 gene expression were higher than control after dietary VD addition, indicating VD treatment enhanced mitochondrial uptake of fatty acyl‐CoA for oxidation in adipose tissue. In addition, the process of fatty acid synthesis was inhibited after VD addition, with the decrease in FAS and ACC gene expression in adipose tissue.

Liver is the center of fatty acid metabolism and lipid circulation in mammals. VD deficiency promotes the progression of fibrosis and causes chronic liver disease (Pop et al. 2022). In our study, VD treatment improved the process of fatty acid oxidation in liver via upregulating CPT1 and CPT2 gene expression, which may be associated with the higher PPAR‐α gene expression. PPAR‐α plays a pivotal role in the metabolism of fatty acids, encompassing both β‐oxidation and ω‐oxidation pathways. Chinetti et al. (2000) found liver had the highest PPAR‐α gene expression level than other tissues, indicating its central importance in lipid homeostasis. Our present results suggest that VD enhances fatty acid oxidation via PPAR‐α in liver, which is in line with the previous study revealing that VD acts as a protective factor in non‐alcoholic fatty liver disease and that VD may alleviate hepatic steatosis via the PPAR‐α signaling pathway (Du et al. 2023).

Fatty acids serve as a crucial fuel substrate for muscular tissues. The process of fatty acid utilization in muscle involves a series of physiological mechanisms, including uptake, intracellular transport, and subsequent oxidation. FATP and FABP, respectively, play roles in the transmembrane transport and intracellular transport of fatty acids, which ensure fatty acids enter cells and metabolism (Choi et al. 2020). Our research indicated that VD administration increased the gene expression of FATP and FABP in skeletal muscle tissue, in line with the previous study (Du et al. 2023), indicating that VD improves the uptake process of fatty acids by muscle cells. In addition, dietary VD addition increased the leg muscle rate, which is similar to the previous study indicating that VD may improve muscle development by accelerating myoblast differentiation.

## 5 Conclusion

Dietary VD addition promotes hair follicle development. Wnt10b/β‐catenin, Notch, SHH, IGF1, and EGF signaling pathways or factors may participate in the regulatory process. Dietary VD addition increases fatty acid oxidation process in fat tissue and liver, and fatty acid uptake in muscle, and inhibits the fatty acid synthesis process in fat tissue. Dietary addition of 1400–2100 IU/kg VD is a better appropriate dose for hair density for Rex rabbits.

## Author Contributions

**Xiang Li:** writing – original draft (equal). **Xiaojing Wu:** methodology (equal). **Fuchang Li:** funding acquisition (equal). **Lei Liu:** funding acquisition (equal), project administration (equal), writing – review and editing (equal).

## Conflicts of Interest

The authors declare no conflicts of interest.

## Data Availability

The authors have nothing to report.
